# Is Body Mass Index a Prognostic Factor in Metastatic HER2-Positive Breast Cancer? A Real-World Multicenter Study

**DOI:** 10.3390/medicina61091604

**Published:** 2025-09-05

**Authors:** Zeliha Birsin, Hülya Odabaşı Bükün, İsmail Nazlı, Onur Alkan, Murat Günaltılı, Emir Çerme, Vali Aliyev, Selin Cebeci, Seda Jeral, Hamza Abbasov, Türkkan Evrensel, Çiğdem Papila, Nebi Serkan Demirci, Özkan Alan

**Affiliations:** 1 Department of Medical Oncology, Cerrahpaşa Faculty of Medicine, Istanbul University–Cerrahpaşa, 34098 Istanbul, Türkiye; murat.gunaltili@iuc.edu.tr (M.G.); emircrm34@gmail.com (E.Ç.); dktr.aliyev@gmail.com (V.A.); sellcebeci@gmail.com (S.C.); sedajeral@gmail.com (S.J.); hamzaabbasov90@gmail.com (H.A.); bpapila@iuc.edu.tr (Ç.P.); drserkannebi@yahoo.com (N.S.D.); ozkan.alan@hotmail.com (Ö.A.); 2 Department of Medical Oncology, Faculty of Medicine, Bursa Uludağ University, 16400 Bursa, Türkiye; hulyaodabasi@uludag.edu.tr (H.O.B.); evrensel@uludag.edu.tr (T.E.); 3 Department of Medical Oncology, Göztepe Prof. Dr. Süleyman Yalçın City Hospital, 34722 Istanbul, Türkiye; mdi.nazli@gmail.com (İ.N.); dronuralkan@gmail.com (O.A.)

**Keywords:** breast cancer, HER2-positive, body mass index, obesity, hormone receptor, progression-free survival, overall survival

## Abstract

*Background and Objectives:* The prognostic significance of body mass index (BMI) in metastatic HER2-positive breast cancer (BC) remains unclear, with previous studies yielding conflicting results. This multicenter real-world study aimed to investigate the prognostic role of BMI in this patient population. *Materials and Methods*: A total of 169 female patients with metastatic HER2-positive BC who received trastuzumab-based treatment between 2010 and 2024 were included. Patients were categorized by BMI (<30 kg/m^2^ vs. ≥30 kg/m^2^). The primary endpoints were overall survival (OS) and progression-free survival (PFS). Kaplan–Meier and Cox regression analyses were performed overall and in subgroups stratified by hormone receptor (HR) status. *Results*: In the overall cohort, a BMI ≥ 30 was not significantly associated with OS or PFS. However, in the HR-positive/HER2-positive subgroup, BMI ≥ 30 kg/m^2^ was linked to significantly shorter OS (*p* = 0.024) and PFS (*p* = 0.047) by Kaplan–Meier analysis. However, these associations did not remain statistically significant in multivariate analyses. No significant BMI-related differences were observed in the HR-negative subgroup. Other independent negative prognostic factors included recurrent disease, the presence of brain metastases, and a high Ki-67 index. *Conclusions*: BMI was not identified as an independent prognostic factor in the overall population. However, among HR-positive/HER2-positive patients, obesity was associated with poorer survival in univariate analysis, but this was not confirmed in multivariate analysis. These findings underscore the need for prospective studies to clarify the prognostic role of adiposity, considering biological subtypes.

## 1. Introduction

BC is the most frequently diagnosed cancer among women and remains the leading cause of cancer-related mortality in this population [1]. It represents a heterogeneous disease with various subtypes characterized by unique molecular profiles, clinical outcomes, and prognostic significance. Around 15–20% of patients with breast cancer exhibit overexpression of the human epidermal growth factor receptor 2 (HER2) [2].

According to the World Health Organization (WHO), a BMI of 30 kg/m^2^ or higher is classified as obesity, whereas a BMI between 25 and 29.9 kg/m^2^ is considered overweight [3]. Overweight and obesity is increasingly recognized as a major global public health challenge [4]. Numerous studies have explored the complex link between BMI and both the risk and prognosis of breast cancer [5,6,7]. In postmenopausal women, a higher BMI is considered an independent risk factor for development BC, whereas it may have a protective effect in premenopausal women [8]. From a pathophysiological perspective, several mechanisms have been proposed to explain how overweight and obesity may influence breast cancer outcomes. These include elevated levels of circulating estrogens, increased insulin and insulin-like growth factors, as well as heightened inflammatory cytokine activity [9,10,11]. BMI has been shown to independently affect overall survival in early-stage breast cancer across all subtypes [12,13,14]. However, in metastatic breast cancer, the relationship between BMI and survival remains unclear, particularly due to the heterogeneity of data regarding its impact in advanced HER2-positive breast cancer [13,15,16,17,18,19]. This real-life multicenter study aimed to evaluate the impact of BMI on clinical outcomes in patients with advanced HER2-positive breast cancer treated with trastuzumab-based therapies.

## 2. Materials and Methods

### 2.1. Study Design and Patient Inclusion Criteria

This multicenter, retrospective real-world cohort was conducted at three oncology centers and included patients treated between January 2010 and June 2024. Eligible participants were female adults (≥18 years) with de novo or recurrent metastatic HER2-positive breast cancer who initiated first-line systemic anti-HER2-based therapy. HER2 positivity was defined as immunohistochemistry (IHC) 3+ or IHC 2+ with confirmed amplification by silver in situ hybridization (SISH). HR status (ER/PR) had to be documented; both HR-positive and HR-negative patients were eligible. The exposure of interest was baseline BMI, calculated as weight (kg)/height (m^2^). BMI had to be measured within 14 days prior to the start of first-line metastatic therapy. Patients were required to have complete baseline clinicopathologic data and outcome data sufficient for time-to-event analyses.

Exclusion criteria were age <18 years; non-metastatic (early-stage) disease; ECOG-PS > 2 at first-line treatment start; unknown or non-amplified HER2 status; no documented height and/or weight within the allowed BMI window; any prior systemic therapy for metastatic disease before the index first-line regimen; follow-up <12 months among survivors (patients who died earlier were retained); or concurrent active second malignancy (except adequately treated non-melanoma skin cancer or in situ cervical carcinoma).

### 2.2. Data Collection, Study Variables, and Outcome Definitions

Clinical, pathological, and treatment-related data were obtained from institutional electronic medical records and archived patient files. Collected variables included demographic characteristics (age, sex), comorbidities, ECOG performance status, BMI, tumor localization (categorized as right, left, or bilateral), disease presentation (de novo or recurrent), histological type and grade, estrogen receptor (ER), progesterone receptor (PR), Ki-67 status, and HER2 expression (defined as IHC 3+ or IHC 2+ with SISH-confirmed amplification). Information regarding metastatic sites and the number of metastatic lesions was also recorded. First-line treatment details included the type of regimen, treatment-related toxicities, and discontinuation if applicable. According to WHO classification, patients were divided into two BMI categories: <30 kg/m^2^ (non-obese) and ≥30 kg/m^2^ (obese). Follow-up data encompassed disease progression, date of last contact, and survival status.

The primary endpoints of the study were overall survival (OS) and progression-free survival (PFS). OS was defined as the time from the diagnosis of metastatic disease to death from any cause. PFS was defined as the time from the initiation of first-line treatment to the date of radiologically confirmed disease progression or death, whichever occurred first. Radiological progression was considered an event for PFS, even if the patient continued on the same treatment following local interventions such as radiotherapy or surgery.

### 2.3. Ethical Considerations

Access to patient data was restricted to the physicians involved in data analysis and manuscript preparation, in accordance with institutional confidentiality policies. The study was conducted in line with institutional and international ethical standards, including the Declaration of Helsinki. Ethical approval was obtained from the Ethics Committee of Istanbul University-Cerrahpaşa, Cerrahpaşa Faculty of Medicine (approval no: 2025/179, dated 5 March 2025).

### 2.4. Statistical Analysis

Statistical analyses were performed using SPSS software, version 26.0 (IBM Corp., Armonk, NY, USA). The distribution of continuous variables was evaluated through the Shapiro–Wilk test, as well as by inspecting Q–Q plots and histograms. Continuous data were expressed as either mean ± standard deviation or median with minimum and maximum values, depending on distribution. Categorical variables were presented as counts and percentages.

For group comparisons, the independent samples t-test or the Mann–Whitney U test was used for continuous variables, depending on normality, while the chi-square test or Fisher’s exact test were used for categorical variables, as appropriate. Survival outcomes were analyzed using the Kaplan–Meier method, with group comparisons assessed by the Log-Rank test. To determine prognostic factors, both univariate and multivariate Cox proportional hazards regression analyses were applied. A two-sided *p*-value of less than 0.05 was considered statistically significant.

## 3. Results

### 3.1. Baseline Clinical, Demographic, and Treatment Characteristics

A total of 169 female patients with metastatic HER2-positive breast cancer were included in the analysis. Patients were stratified by BMI into two groups: <30 kg/m^2^ (*n* = 109) and ≥30 kg/m^2^ (*n* = 60). The median age of the entire cohort was 51 years (range: 26–84), with patients in the higher BMI group being significantly older than those in the lower BMI group (median: 56 vs. 49 years, *p* = 0.019). Postmenopausal status and the presence of comorbidities were more common in the BMI ≥30 group (*p* = 0.024 and *p* = 0.009, respectively). No significant differences were noted between two groups in terms of histological subtype, hormone receptor status, Ki-67 index, and tumor grade. The majority of tumors were invasive ductal carcinoma (83%) and HR-positive (66%). Regarding metastatic presentation, 63% of patients had de novo metastatic disease, and 24% had bone-only metastasis. Visceral involvement and brain metastasis rates did not differ significantly between BMI groups.

In terms of first-line treatment, the majority of patients received a THP regimen (trastuzumab + pertuzumab + taxane), with similar distribution across both BMI groups (*p* = 0.982). At the time of last follow up, there was a trend toward higher mortality in the BMI ≥ 30 group compared to the BMI < 30 group (46% vs. 32%), although this difference did not reach statistical significance (*p* = 0.061). Baseline demographic, clinical and treatment characteristics of the study population are outlined in Table 1.

### 3.2. Progression-Free Survival Outcome

**Figure 1:**
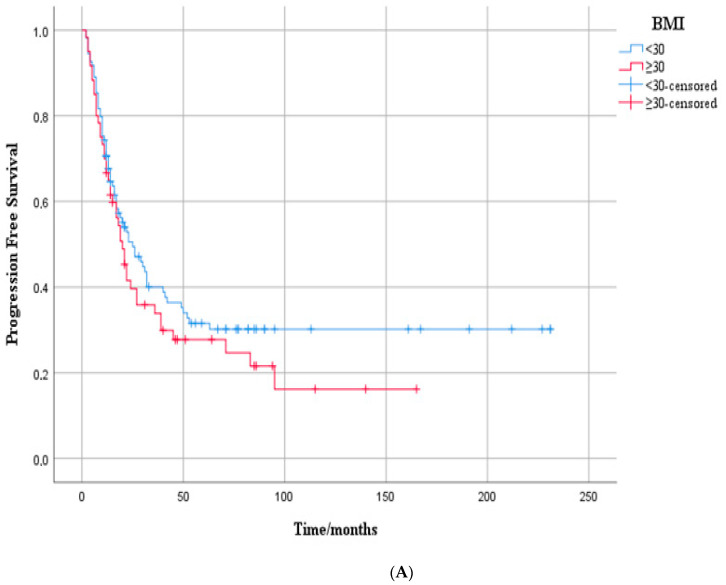

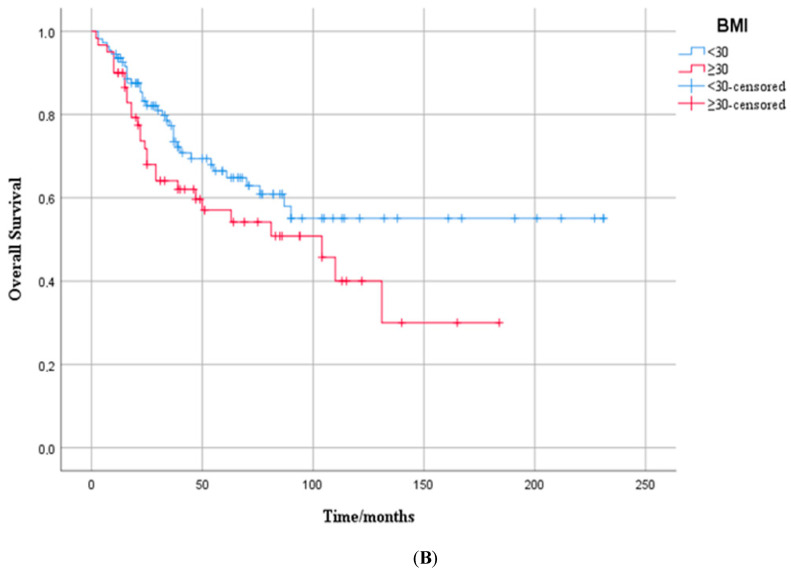
(**A**) Kaplan–Meier curves according to BMI (<30 vs. ≥30 kg/m^2^) in the overall cohort of metastatic HER2-positive breast cancer patients: progression-free survival. Progression-free survival by BMI group. Kaplan–Meier curves showing PFS in patients with BMI < 30 (blue) and BMI ≥30 (red). Median PFS was 30.0 months (95% CI: 17.1–42.9) vs. 21.0 months (95% CI: 14.0–28.0), respectively. Although PFS was shorter in the higher BMI group, the difference was not statistically significant (log-rank *p* = 0.148). Censored cases are marked with plus symbols. (**B**) Kaplan–Meier curves according to BMI (<30 vs. ≥30 kg/m^2^) in the overall cohort of metastatic HER2-positive breast cancer patients’ overall survival. Overall survival by BMI group. Kaplan–Meier curves showing OS in patients with BMI < 30 (blue) and BMI ≥ 30 (red). Median OS was 81.0 months (95% CI: 23.7–138.3) in the higher BMI group, while it was not reached in the lower BMI group. Although OS appeared longer in patients with BMI < 30, the difference was not statistically significant (log-rank *p* = 0.111). Censored cases are marked with plus symbols.

In the overall cohort, the median PFS was 24.0 months (95% CI: 17.5–30.5). The median PFS was 30.0 months (95% CI: 17.1–42.9) in patients with BMI < 30 kg/m^2^, and 21.0 months (95% CI: 14.0–28.0) in those with BMI ≥ 30 kg/m^2^. Although the PFS was numerically shorter in the higher BMI group, this difference did not reach statistical significance (log-rank *p* = 0.148) (Figure 1A).

In the univariate analysis, recurrent disease, bone-only metastasis, the presence of brain metastases and higher Ki-67 index were significantly associated with PFS. In the multivariate Cox regression analysis, four variables remained independent predictors of PFS. Patients with recurrent disease had significantly shorter PFS compared to those with de novo presentation (HR: 2.28; 95% CI: 1.46–3.55; *p* < 0.001). Bone-only metastasis was associated with longer PFS (HR: 0.60; 95% CI: 0.36–1.82; *p* = 0.047), whereas the presence of brain metastases predicted shorter PFS (HR: 2.22; 95% CI: 0.24–0.83; *p* = 0.011). Additionally, a higher Ki-67 proliferation index was independently associated with an increased risk of disease progression (HR: 1.01; 95% CI: 1.00–1.03; *p* = 0.01). BMI showed no significant association with PFS in either univariate or multivariate analysis (Table 2).

**Figure 2:**
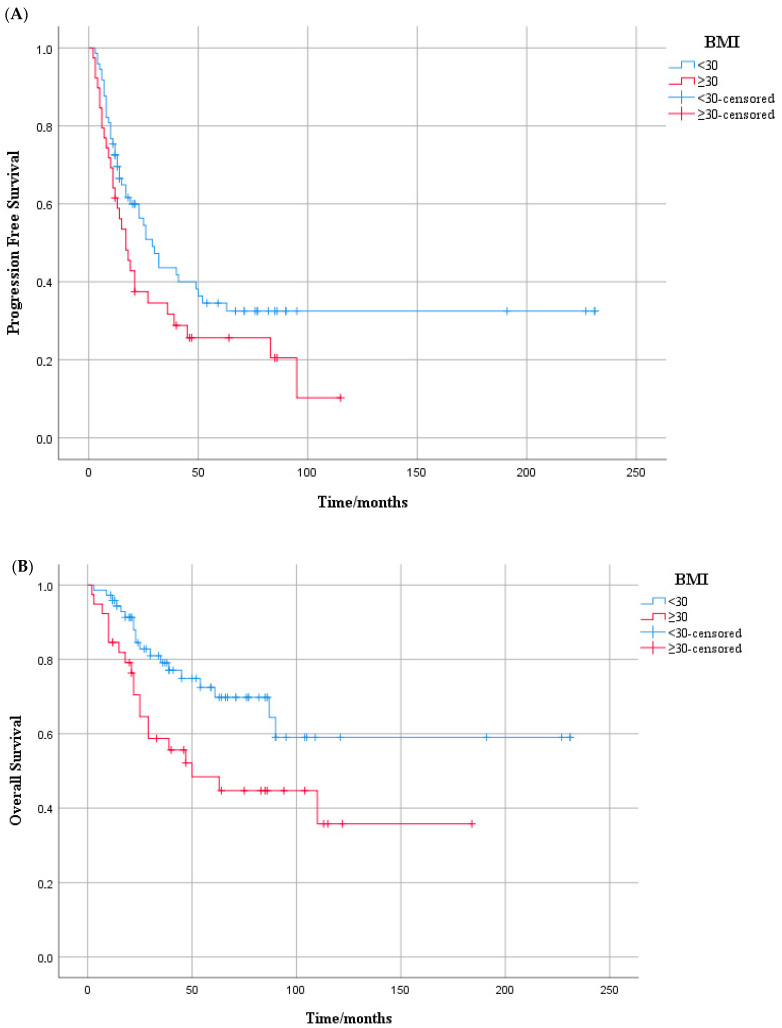
Kaplan–Meier curves according to BMI (<30 vs. ≥30 kg/m^2^) in the HR-positive/HER2-positive subgroup of metastatic breast cancer patients: (**A**) progression-free survival, (**B**) overall survival. (**A**) Progression-free survival in HR-positive/HER2-positive patients by BMI group. Kaplan–Meier curves showing PFS in HR+/HER2+ patients with BMI < 30 (blue) and BMI ≥ 30 (red). Median PFS was 32.0 months (95% CI: 6.4–57.6) vs. 17.0 months (95% CI: 8.5–25.5), respectively. The difference was statistically significant (log-rank *p* = 0.047). Censored cases are marked with plus symbols. (**B**) Overall survival in HR-positive/HER2-positive patients by BMI group. Kaplan–Meier curves showing OS in HR+/HER2+ patients with BMI < 30 (blue) and BMI ≥ 30 (red). Median OS was 61.0 months (95% CI: 32.3–89.7) in the higher BMI group, while it was not reached in the lower BMI group. This difference was statistically significant (log-rank *p* = 0.024). Censored cases are marked with plus symbols.

When we evaluated subgroups based on hormone receptor status, BMI appeared to have a differential impact on PFS. Among patients with HR-positive/HER2-positive tumors, those with a BMI < 30 kg/m^2^ had a median PFS of 32.0 months (95% CI: 6.4–57.6), compared to 17.0 months (95% CI: 8.5–25.5) in the BMI ≥ 30 group, with this difference reaching statistical significance (log-rank test, *p* = 0.047) (Figure 2A). In contrast, for patients with HR-negative/HER2-positive disease, median PFS was 20.0 months (95% CI: 11.9–28.1) in the lower BMI group and 24.0 months (95% CI: 13.1–34.9) in the higher BMI group, with no significant difference observed between the groups (log-rank test, *p* = 0.819) (Figure S1A). In the HR-positive/HER2-positive subgroup, higher BMI (≥30 kg/m^2^) was associated with a trend toward shorter PFS in the univariate analysis (HR: 1.60; 95% CI: 0.99–2.55; *p* = 0.051), but this was not statistically significant in the multivariate model (HR: 1.48; 95% CI: 0.89–2.46; *p* = 0.127) (Table S1).

### 3.3. Overall Survival Outcome

In the overall cohort, the median OS was 110 months (95% CI: not reached). The median OS was 81.0 months (95% CI: 23.7–138.3) in patients with BMI ≥ 30 kg/m^2^. The median OS for patients with BMI < 30 kg/m^2^ could not be reached. Although OS appeared longer in the lower BMI group, this difference did not reach statistical significance (log-rank *p* = 0.111) (Figure 1B).

In univariate analysis, higher BMI was associated with a trend toward worse overall survival (HR: 1.53; 95% CI: 0.93–2.52; *p* = 0.092), but this association did not reach significance in the multivariate model (HR: 1.43; 95% CI: 0.84–2.45; *p* = 0.181). A higher Ki-67 index was independently associated with a poorer OS (HR: 1.01; 95% CI: 1.00–1.03; *p* = 0.01). Patients with recurrent disease had significantly worse OS compared to those with de novo presentation (HR: 1.79; 95% CI: 1.05–3.07; *p* = 0.032). Bone-only metastasis was significant in univariate analysis (HR: 0.36; *p* = 0.007), but not in the multivariate model (*p* = 0.092). The presence of brain metastases remained a strongly independent negative prognostic factor (HR: 2.23; 95% CI: 0.24–0.83; *p* = 0.011) (Table 3).

When subgroup analyses were performed according to hormone receptor status, a significant OS difference was observed in the HR-positive/HER2-positive group. Patients with BMI ≥ 30 kg/m^2^ had a median OS of 61.0 months (95% CI: 32.3–89.7), whereas the median OS was not reached in the BMI < 30 group (*p* = 0.024, log-rank test) (Figure 2B). In contrast, among HR-negative/HER2-positive patients, no significant difference in OS was found between BMI groups (median OS: 76.0 vs. 104.0 months; *p* = 0.740, log-rank test) (Figure S1B).

In the HR-positive/HER2-positive subgroup, a higher BMI (≥30 kg/m^2^) was significantly associated with worse OS in univariate analysis (HR: 2.11; 95% CI: 1.12–3.97; *p* = 0.02), but this association did not remain significant after adjustment for the multivariate model (HR: 1.56; 95% CI: 0.80–3.03; *p* = 0.186). Among other variables, a higher Ki-67 index (HR: 1.02; 95% CI: 1.00–1.03; *p* = 0.047) and recurrent metastatic presentation (HR: 2.22; 95% CI: 1.17–4.18; *p* = 0.014) were identified as independent negative prognostic factors for OS (Table S2).

## 4. Discussion

In this multicenter real-world study including 169 patients with metastatic HER2-positive breast cancer treated with trastuzumab-based therapies, the prognostic impact of BMI was evaluated. While a higher BMI (≥30 kg/m^2^) was associated with numerically shorter PFS and OS in the overall cohort, these differences were not statistically significant. Notably, in the HR-positive/HER2-positive subgroup, Kaplan–Meier analysis revealed that patients with BMI ≥30 kg/m^2^ had significantly shorter PFS and OS compared to those with BMI < 30 kg/m^2^. Despite these findings, BMI did not emerge as an independent prognostic factor in multivariate analyses. Recurrent disease, brain metastases, and higher Ki-67 index were consistently associated with worse clinical outcomes.

The “obesity paradox,” which refers to the association between higher BMI and improved overall survival, has been described in several cancer types [20,21]. In contrast, various meta-analyses have demonstrated that obesity is associated with poorer outcomes in patients with breast cancer [22,23]. However, when focusing on metastatic HER2-positive breast cancer, the findings in the literature remain inconsistent. In a multicenter study, Krasniqi et al. reported that patients with BMI ≥ 30 kg/m^2^ had significantly worse OS, while no association was observed with PFS [17]. In the meta-analysis conducted by Lohmann et al., obesity was found to be associated with worse DFS and OS across all breast cancer subtypes, including HER2-positive tumors [14]. On the other hand, in a study by Modi and colleagues involving patients with metastatic HER2-positive breast cancer, elevated BMI was associated with improved PFS and OS, supporting the presence of an obesity paradox in the advanced disease setting [13]. There are similar studies conducted across all metastatic breast cancer subtypes that support this finding [16,24]. Apart from these, in the study conducted by Martel and colleagues, BMI was not found to significantly influence PFS or OS in patients with HER2-positive metastatic breast cancer [15]. Our results are consistent with this study, as BMI did not have a statistically significant impact on OS or PFS in our cohort.

Lohmann and colleagues evaluated HER2-positive breast cancer by stratifying patients according to HR status. Their analysis revealed no statistically significant prognostic differences between HR-positive and HR-negative subgroups in terms of BMI-related outcomes [14]. Mazzarella and colleagues analyzed 1250 patients with early-stage HER2-positive breast cancer and found that obesity (BMI ≥ 30) was significantly associated with a worse OS and a higher incidence of distant metastases in the ER-negative subgroup. In contrast, BMI did not appear to influence outcomes in ER-positive patients [25]. In contrast to these findings, our study demonstrated that while BMI did not significantly impact OS or PFS in the overall cohort, in the HR-positive HER2-positive subgroup, patients with BMI ≥ 30 kg/m^2^ had worse PFS and OS. However, BMI was not identified as an independent prognostic factor in this subgroup according to multivariate Cox regression analysis. In the hormone-receptor-negative subgroup, obesity was not associated with a significant difference in either OS or PFS. In HR-positive/HER2-positive breast cancer patients, both ERα and HER2 signaling pathways are active and interact with each other [26]. In overweight or obese patients, increased aromatase activity in adipose tissue may lead to elevated estradiol levels [27], potentially contributing to poorer outcomes in this subgroup.

In conclusion, obesity may exert a dual and complex role in cancer progression and outcomes. On one hand, excess BMI may offer a protective buffer against cancer-associated cachexia, potentially contributing to improved overall survival in certain patients [20,21]. On the other hand, obesity is also associated with chronic low-grade inflammation, insulin resistance, altered adipokine profiles—including increased leptin and decreased adiponectin—and changes in drug pharmacokinetics, all of which may negatively affect cancer prognosis [14]. Also, the link between obesity and increased cancer risk and worse outcomes may be partly explained by Regulated in Development and DNA Damage Responses 1 (REDD1), which downregulates mTORC1 [28] and is induced by obesity [29]; its overexpression correlates with tumor survival, invasion/metastasis, treatment resistance, and poorer prognosis across multiple malignancies, including breast cancer, pointing to an mTOR-centered mechanistic pathway [30,31,32,33].

Building on this framework, we investigated whether baseline BMI is independently associated with outcomes. At this point, the novelty of our work lies in its focus on a real-world, multicenter cohort of metastatic HER2-positive disease, explicitly evaluating HR-specific effects and testing whether any BMI outcome signal persists after multivariable adjustment for clinical factors. Our findings suggest that the prognostic impact of obesity in breast cancer may vary across molecular subtypes. In the metastatic setting, our study may contribute by providing evidence that BMI alone may be insufficient as an independent prognostic marker once these factors are accounted for. Taken together, these results may help refine the field’s understanding of the “obesity paradox” and suggest that future work could integrate molecular subtype, body composition measures beyond BMI (e.g., visceral adiposity, sarcopenia), and longitudinal weight trajectories when evaluating adiposity–outcome relationships.

This study has several limitations. Its retrospective design restricts causal interpretations. BMI was assessed only at the time of metastatic diagnosis, without accounting for changes during treatment. Additionally, lack of data on body composition (such as fat and muscle distribution) limited a more detailed evaluation of adiposity. The relatively small sample size in some subgroups may have reduced statistical power, and variability in treatment regimens could have influenced survival outcomes.

## 5. Conclusions

This real-world multicenter study suggests that BMI is not an independent prognostic factor in patients with metastatic HER2-positive breast cancer overall. However, in the HR-positive subgroup, higher BMI may be associated with worse survival outcomes. These findings highlight the complex interplay between obesity and tumor biology, emphasizing the need for further prospective research that incorporates body composition analysis and molecular subtype stratification.

## Figures and Tables

**Table 1:** Clinicopathological characteristics of metastatic HER2-positive breast cancer patients stratified by BMI categories (<30 vs. ≥30 kg/m^2^).

Variables		Overall Cohort (*n* = 169)		BMI < 30 (*n* = 109)		BMI ≥ 30 (*n =* 60)		*p*
Age, median		51 (min 26–max 84)		49 (min 26–max 84)		56 (min 28–max 78)		0.019 ^1^
Menopause status, *n* (%)	Premenopausal	76	45%	56	52%	20	33%	0.024 ^2^
	Postmenopausal	93	55%	53	49%	40	66%	
Comorbidity, *n* (%)	Absent	82	49%	61	56%	21	34%	0.009 ^2^
	Present	87	51%	48	44%	39	64%	
ECOG, *n* (%)	0	129	76%	90	83%	39	64%	0.010 ^2^
	1	40	24%	19	18%	21	34%	
BMI kg/m^2^, median		27.8 (min 19.7–max 46.8)		26.1 (min 19.7–max 29.9)		32.8 (min 30–max 46.8)		0.000 ^1^
Primary tumor laterality, *n* (%)	Right	84	50%	56	52%	28	46%	0.558 ^2^
	Left	85	50%	53	49%	32	52%	
Histological subtype, *n* (%)	IDC	140	83%	93	86%	47	77%	0.365 ^2^
	ILC	7	4%	4	4%	3	5%	
	Mixed-other	22	13%	12	10%	10	18%	
ER, median		40 (min 0–max 100)		40 (min 0–max 100)		25 (min 0–max 100)		0.984 ^1^
PR, median		1 (min 0–max 100)		1 (min 0–max 100)		0 (min 0–max 95)		0.638 ^1^
Ki-67%, median		35 (min 5–max 90)		35 (min 5–max 90)		40 (min 5–max 90)		0.218 ^1^
Grade, *n* (%)	2	64	38%	45	42%	19	31%	0.217 ^2^
	3	105	62%	64	59%	41	67%	
Subtype, n (%)	HR-negative	57	34%	36	33%	21	34%	0.795 ^2^
	HR-positive	112	66%	73	68%	39	64%	
Pattern of metastatic presentation, *n* (%)	De novo	107	63%	74	69%	33	54%	0.096 ^2^
	Recurrent	62	37%	35	32%	27	44%	
Bone-only disease		40	24%	24	22%	16	26%	0.496 ^2^
Visceral metastasis, *n* (%)		97	57%	60	56%	37	61%	0.405 ^2^
Brain metastasis, *n* (%)		25	15%	16	15%	9	15%	0.955 ^2^
First-line systemic therapy	THP	100	59%	64	59%	36	59%	0.982 ^2^
	TH	60	36%	39	36%	21	34%	
	T-DM1	9	5%	6	6%	3	5%	
Current status	Alive	106	63%	74	69%	32	52%	0.061 ^2^
	Deceased	63	37%	35	32%	28	46%	

^1^ Mann–Whitney U test; ^2^ chi-squared test; BMI: body mass index; IDC: invasive ductal carcinoma; ILC: invasive lobular carcinoma; ER: estrogen receptor; PR: progesterone receptor; THP: trastuzumab + pertuzumab + taxane; TH: trastuzumab + taxane; T-DM1: ado-trastuzumab emtansine.

**Table 2:** Univariate and multivariate Cox regression analysis for progression-free survival in patients with metastatic HER2-positive breast cancer.

Variables		Univariate Analysis		Multivariate Analysis	
		HR (95% CI)	*p*	HR (95% CI)	*p*
Age		0.99 (0.97–1.00)	0.305		
Comorbidity	Absent	0.76 (0.52–1.11)	0.160	1.41 (0.96–2.06)	0.083
	Present				
BMI	<30	1.24 (0.85–1.81)	0.260		
	≥30				
Menopause status	Premenopausal	0.72 (0.50–1.05)	0.095	1.24 (0.80–1.91)	0.328
	Postmenopausal				
Grade	2	0.95 (0.65–1.39)	0.800		
	3				
ER		1.00 (0.99–1.00)	0.839		
PR		1.00 (0.99–1.00)	0.747		
Ki-67		1.00 (0.99–1.01)	0.125	1.01 (1.00–1.03)	0.01
Pattern of metastatic presentation	De novo	2.20 (1.51–3.19)	0.000	2.28 (1.46–3.55)	0.000
	Recurrent				
Subtype	HR-negative	0.95 (0.64–1.40)	0.807		
	HR-positive				
Visceral metastasis	Absent	1.16 (0.80–1.70)	0.420		
	Present				
Bone-only disease	Absent	0.51 (1.20–3.20)	0.007	0.60 (0.36–1.82)	0.047
	Present				
Brain metastasis	Absent	2.51 (1.57–3.99)	0.000	2.22 (0.24–0.83)	0.011
	Present				

BMI: body mass index; ER: estrogen receptor; PR: progesterone receptor.

**Table 3:** Univariate and multivariate Cox regression analysis for overall survival in patients with metastatic HER2-positive breast cancer.

Variables		Univariate Analysis		Multivariate Analysis	
		HR (95% CI)	*p*	HR (95% CI)	*p*
Age		1.01 (0.98–1.02)	0.416		
Comorbidity	Absent	0.702 (0.42–1.15)	0.165		
	Present				
BMI	<30	1.53 (0.93–2.52)	0.092	1.43 (0.84–2.45)	0.181
	≥30				
Menopause status	Premenopausal	0.79 (0.48–1.29)	0.350		
	Postmenopausal				
Grade	2	1.11 (0.66–1.85)	0.068		
	3				
ER		1.01 (0.99–1.00)	0.439		
PR		1.03 (0.99–1.00)	0.902		
Ki-67		1.02 (1.01–1.03)	0.000	1.01 (1.00–1.03)	0.01
Pattern of metastatic presentation	De novo	2.62 (1.59–4.32)	0.000	1.79 (1.05–3.07)	0.032
	Recurrent				
Subtype	HR-negative	1.11 (0.66–1.85)	0.682		
	HR-positive				
Visceral metastasis	Absent	1.16 (0.70–1.92)	0.554		
	Present				
Bone-only disease	Absent	0.36 (1.31–5.85)	0.007	0.51 (0.89–4.19)	0.092
	Present				
Brain metastasis	Absent	3.79 (2.16–6.65)	0.000	2.23 (0.24–0.83)	0.011
	Present				

BMI: body mass index; ER: estrogen receptor; PR: progesterone receptor.

## Data Availability

The data supporting the findings of this study are available from the corresponding author upon reasonable request.

## Supplementary Materials

The following supporting information can be downloaded at https://www.mdpi.com/article/10.3390/medicina61091604/s1: Figure S1: Kaplan–Meier curves according to BMI (<30 vs. ≥30 kg/m^2^) in the HR-negative/HER2-positive subgroup of metastatic breast cancer patients: (A) progression-free survival, (B) overall survival; Table S1: univariate and multivariate Cox regression analysis for progression-free survival in patients with metastatic HR-positive HER2-positive breast cancer; Table S2: Univariate and multivariate Cox regression analysis for overall survival in patients with metastatic HR-positive HER2-positive breast cancer.

## Abbreviations

The following abbreviations are used in this manuscript:

BC	Breast cancer
BMI	Body mass index
CI	Confidence interval
ECOG-PS	Eastern Cooperative Oncology Group performance status
ER	Estrogen receptor
ERα	Estrogen receptor alpha
HER2	Human epidermal growth factor receptor 2
HR (hormone receptor)	ER and/or PR status
HR (hazard ratio)	Effect estimate from Cox models
IHC	Immunohistochemistry
IDC	Invasive ductal carcinoma
ILC	Invasive lobular carcinoma
ISH	In situ hybridization
KM	Kaplan–Meier
mTORC1	Mechanistic target of rapamycin complex 1
OS	Overall survival
PFS	Progression-free survival
PR	Progesterone receptor
REDD1	Regulated in Development and DNA Damage Responses 1
SISH	Silver in situ hybridization
SPSS	Statistical Package for the Social Sciences
T-DM1	Ado-trastuzumab emtansine
TH	Trastuzumab + taxane
THP	Trastuzumab + pertuzumab + taxane
WHO	World Health Organization
