# Supplementary material for: Is Body Mass Index a Prognostic Factor in Metastatic HER2-Positive Breast Cancer? A Real-World Multicenter Study
Source: Medicina (Kaunas). 2025 Sep 5;61(9):1604. doi: 10.3390/medicina61091604 (PMC12471550; doi:10.3390/medicina61091604)
Supplement: Supplementary file 1 [file medicina-61-01604-s001.zip › medicina-3826030-supplementary.pdf]

Supplementary file

Table S1. Univariate and Multivariate Cox Regression Analysis for Progression Free Survival in Patients with Metastatic HR-positive HER2-Positive Breast Cancer					
**Variables**		Univariate Analiz		Multivariate Analiz	
		HR (95%CI)	p	HR (95%CI)	P
Age		1.00(0.99-1.02)	0.442		
Comorbidty	Absent	1.09(0.69-1.74)	0.698		
	Present				
BMI	<30	1.60(0.99-2.55)	0.051	1.48(0.89-2.46)	0.127
	≥30				
Menopause status	Premenopausal	1.14(0.72-1.82)	0.557		
	Postmenopausal				
Grade	2	0.99(0.65-1.58)	0.329		
	3				
ER		1.00(0.99-1.01)	0.614		
PR		1.00(0.99-1.00)	0.767		
Ki-67		1.05(0.99-1.01)	0.523		
Pattern of metastatic presentation	De novo	2.05(1.28-3.27)	0.003	2.24(1.31-3.83)	0.003
	Recurrent				
Visceral metastasis	Absent	1.10(0.69-1.76)	0.676		
	Present				
Bone only disease	Absent	1.50(0.88-2.57)	0.134	1.26(0.72-2.73)	0.404
	Present				
Brain metastasis	Absent	1.96(1.00-3.84)	0.049	1.24(0.24-0.83)	0.134
	Present				
BMI: Body Mass Index; ER: Estrogen Receptor; PR: Progesterone Receptor					

Table S2. Univariate and Multivariate Cox Regression Analysis for Overall Survival in Patients with Metastatic HR-Positive HER2-Positive Breast Cancer					
**Variables**		Univariate Analiz		Multivariate Analiz	
		HR (95%CI)	p	HR (95%CI)	P
Age		1.02(0.99-1.04)	0.092	1.01(0.98-1.03)	0.449
Comorbidty	Absent	0.92(0.49-1.73)	0.804		
	Present				
BMI	<30		0.020		0.186

	≥30	2.11(1.12-3.97)		1.56(0.80-3.03)	
Menopause status	Premenopausal	1.11(0.59-2.09)	0.734		
	Postmenopausal				
Grade	2	0.93(0.49-1.75)	0.823		
	3				
ER		1.01(1.00-1.02)	0.059	1.01(1.00-1.02)	0.057
PR		1.00(0.99-1.01)	0.608		
Ki-67		1.02(1.00-1.04)	0.009	1.02(1.00-1.03)	0.047
Pattern of metastatic presentation	De novo	2.32(1.23-4.35)	0.009	2.22(1.17-4.18)	0.014
	Recurrent				
Visceral metastasis	Absent	1.28(0.67-2.45)	0.445		
	Present				
Bone only disease	Absent	2.06(0.86-4.93)	0.104	2.04(0.85-3.46)	0.108
	Present				
Brain metastasis	Absent	2.22(0.97-5.07)	0.058	1.40(0.56-0.83)	0.464
	Present				

BMI: Body Mass Index; ER: Estrogen Receptor; PR: Progesterone Receptor

Figure S1. Kaplan–Meier Curves According to BMI (<30 vs. ≥30 kg/m²) in the HR-Negative/HER2-Positive Subgroup of Metastatic Breast Cancer Patients: (A) Progression-Free Survival, (B) Overall Survival

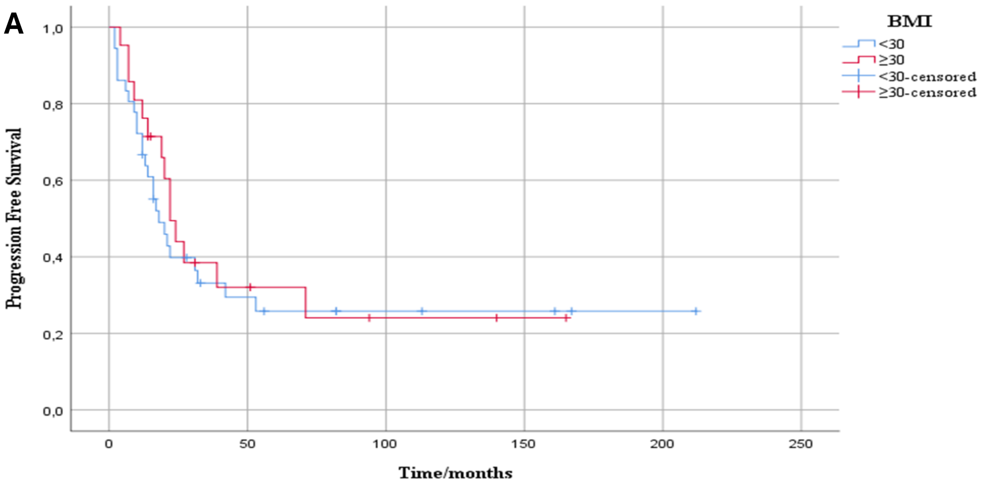

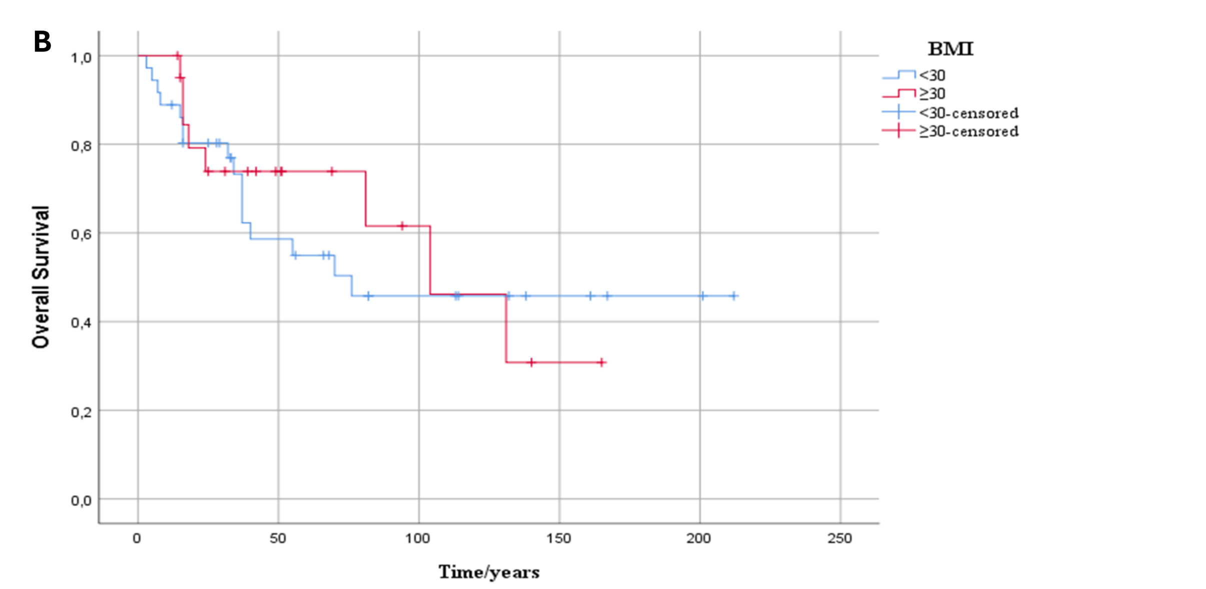

Figure S1. Kaplan–Meier curves for progression-free survival (A) and overall survival (B) by BMI (<30 vs. ≥30 kg/m²) in the HR-negative/HER2-positive subgroup.
